# Preoperative statin therapy and infectious complications in cardiac surgery

**DOI:** 10.1007/s12471-014-0581-5

**Published:** 2014-07-24

**Authors:** N. L. Hartholt, T. C. D. Rettig, M. Schijffelen, W. J. Morshuis, E. M. W. van de Garde, P. G. Noordzij

**Affiliations:** 1 Department of Clinical Pharmacy, St. Antonius Hospital, Koekoekslaan 1, 3430 EM Nieuwegein, the Netherlands; 2 Department of Anesthesiology, Intensive Care and Pain Medicine, St. Antonius Hospital, Koekoekslaan 1, 3430 EM Nieuwegein, the Netherlands; 3 Department of Medical Microbiology and Immunology, St. Antonius Hospital, Koekoekslaan 1, 3430 EM Nieuwegein, the Netherlands; 4 Department of Cardiothoracic Surgery, St. Antonius Hospital, Koekoekslaan 1, 3430 EM Nieuwegein, the Netherlands

**Keywords:** Statins, Postoperative infection, Cardiac surgery, Surgical site infection

## Abstract

**Aim:**

To assess whether preoperative statin therapy is associated with the risk of postoperative infection in patients undergoing cardiac surgery.

**Methods:**

520 patients undergoing cardiac surgery in 2010 were retrospectively examined. Data regarding statin and antibiotic use prior to and after surgery were available from the hospital pharmacy information system. Cultures and clinical data of patients on postoperative antibiotics other than standard prophylactic therapy were studied to identify postoperative infections up to 30 days from day of surgery.

**Results:**

370 (71.2 %) patients were on preoperative statin therapy. Overall, 82 patients (15.8 %) suffered from postoperative infection of which 11 were surgical site infections. In multivariable regression analysis, statin therapy was associated with a reduced risk of postoperative infection (adjusted odds ratio: 0.329, 95 %: CI 0.19–0.57; *P* < 0.001).

**Conclusions:**

Preoperative statin use was associated with a considerable reduced risk of postoperative infections following cardiac surgery. Randomised controlled trials are required to clarify the role of statin therapy in the prevention of postoperative infections.

## Introduction

Annually, approximately 16,000 patients are scheduled for cardiothoracic surgery in the Netherlands. [1] Postoperative infections are important causes of increased length of hospital stay and mortality. Despite prophylactic antibiotic therapy, the incidence of postoperative infectious complications is 5 to 21 % [2–4].

In a search for additional ways to decrease the postoperative infection rate, statin therapy is gaining increasing attention. In addition to cholesterol-lowering characteristics, statins have anti-inflammatory, immunomodulatory, antioxidant and antiapoptotic effects that could alter the response to infections. [5] Whether statins have a beneficial effect on postoperative infectious complications in patients undergoing cardiac surgery is unknown, as prior studies have shown conflicting results [6–8].

This study aims to assess whether preoperative statin therapy is associated with the risk of postoperative infection in patients undergoing cardiac surgery.

## Methods

### Study population

The total population of 1868 adult patients who had undergone cardiac surgery in the St. Antonius Hospital, Nieuwegein, the Netherlands in 2010 was the target population for this study. From this population we selected all patients with data on preoperative drug use in the hospital pharmacy information system. This means that only patients who were hospitalised more than 24 h before the day of surgery could be studied (*n* = 593), as these are the patients with reviewed preoperative medication profiles available in the hospital pharmacy information system. Additionally, 73 patients were excluded as they were on antimicrobial therapy prior to their surgical procedure outside the routine antimicrobial prophylaxis (cefazolin 2 g intravenously before skin incision followed by additional cefazolin every four hours for the duration of the procedure and continued up to 48 h for patients with valve or aortic surgery with exogenous materials). Finally, all patients with a statin in the preoperative medication profile were considered exposed at the time of surgery and all other patients were classified as controls. The local Medical Ethics Committee (Research and Development Department, St. Antonius Hospital) approved the study.

### Outcome assessment

The primary outcome was postoperative infection within 30 days of surgery. We used the following approach to determine if a postoperative infection was present: first, all patients on postoperative antibiotic therapy, aside from perioperative antibiotic prophylaxis, were identified. Secondly, N.H. and T.R. independently examined the medical records of these patients to determine if clinical data (e.g. fever, white cell count) supported the diagnosis of infection. When N.H. and T.R. agreed that a postoperative infection was present, it was classified as a surgical site or non-surgical site infection. Subsequently, M.S. and T.R. used microbiological data to identify the microbial aetiology. When N.H., T.R. and M.S. could not reach an agreement on whether an infection was present or on the site of infection, P.N. was consulted. For all patients, in-hospital mortality was noted.

### Potential confounders

To be able to control for potential confounding, medical information was obtained on risk factors for infection that could potentially confound the association between statin treatment and outcome. For each patient we evaluated the presence of the following comorbidities as potential confounders: diabetes, chronic obstructive pulmonary disease (COPD, graded according to the GOLD classification [9] where all smokers were considered class I unless otherwise stated), peripheral artery disease, three-vessel coronary artery disease, renal insufficiency (defined as serum creatinine >120 μmol/l), heart failure (according to New York Heart Association functional classification), prior myocardial infarction, and history of cardiac surgery. In addition, the following surgery-related parameters were assessed: type of surgery (coronary artery bypass graft (CABG), valve surgery, aortic surgery or other), urgent procedure (patients not electively admitted for operation who require surgery before hospital discharge), perfusion time, aortic clamp time and whether rethoracotomy has taken place. Aspirin, antidiabetic agents, beta-blockers, angiotensin-converting enzyme (ACE) inhibitors, proton pump inhibitors (PPIs) and prednisone were examined as possible confounding drugs. A patient was considered exposed to a drug if it was listed in the preoperative medication profile. Furthermore, we calculated the body mass index and European System for Cardiac Operative Risk Evaluation (EURO) score as proxy for overall health status and complication risk. During the study period, glucose values were standardly targeted at 4.5–8 mmol/l during surgery and ICU admission, and 4.5–10 mmol/l on the surgical ward in all patients.

### Statistical analysis

Data were analysed using International Business Machines Statistical Package for the Social Science (IBM SPSS) Statistics 19.0 software. Univariate analysis by *χ* ^2^ tests and Student’s t tests was used to test for significant differences in characteristics between statin users and non-users and patients with and without postoperative infections. Multivariable logistic regression analysis was used to estimate the strength of the association between statin treatment and the risk of postoperative infection and expressed as odds ratios (OR) with 95 % confidence intervals (CI). First, we included all potential confounders and age and gender in a multivariable model if a variable was imbalanced (*P* < 0.10) related to either statin use or occurrence of infection. Second, a final model was obtained after stepwise backward elimination of potential confounders if they did not alter the OR by more than 10 %. Stratified analyses were conducted to detect possible differences in effects related to timing of statin exposure (continued use, commenced use or discontinued use). For all tests, a *P*-value of 0.05 was considered significant.

## Results

A total of 370 (71.2 %) patients received statin treatment until the day of surgery, while 150 (28.8 %) patients did not. Patients receiving statin treatment were more likely to be male, and suffering from COPD, diabetes, peripheral artery disease, and three-vessel coronary artery disease. Furthermore, they experienced more myocardial infarction and isolated CABG surgery and received more frequent other cardiovascular drug therapies, such as aspirin, beta-blockers and ACE-inhibitors. Compared with non-users, patients on statin therapy had shorter perfusion times and aortic clamp times. Baseline characteristics are shown in Table 1.

**Table 1:** Patient characteristics according to the use of statins

Variable	Statin group (*n* = 370)	Control group (*n* = 150)	*p*-value
Patient characteristics			
Sex (male)	260 (70.3)	87 (58.0)	0.007
Age (years)	68.3 ± 10.0	69.5 ± 13.7	0.277
BMI (kg/m^2^)	27.7 ± 4.5	26.7 ± 4.9	0.037
COPD	206 (55.7)	70 (46.7)	0.062
Diabetes	112 (30.3)	27 (18.0)	0.004
Congestive heart failure	140 (37.8)	75 (50.0)	0.011
Renal failure	56 (15.1)	28 (18.7)	0.322
Peripheral artery disease	79 (21.4)	19 (12.7)	0.018
Three-vessel coronary artery disease	209 (56.5)	30 (20.0)	<0.001
EURO score	5.2 ± 3.7	5.7 ± 3.3	0.106
Previous myocardial infarction	180 (48.6)	24 (16.0)	<0.001
Previous cardiac surgery	39 (10.5)	23 (15.3)	0.127
Preoperative medication			
Beta-blocker	293 (79.2)	91 (60.7)	<0.001
ACE-inhibitor	163 (44.1)	47 (31.3)	0.007
Prednisone	43 (11.6)	16 (10.7)	0.756
Proton pump inhibitor	161 (43.5)	60 (40.0)	0.463
Aspirin	150 (40.5)	23 (15.3)	<0.001
Postoperative medication			
Statin	349 (94.3)	37 (24.7)	<0.001
Beta-blocker	301 (81.4)	110 (73.3)	0.056
ACE-inhibitor	168 (45.4)	68 (45.3)	0.968
Prednisone	35 (9.5)	21 (14.0)	0.126
Proton pump inhibitor	272 (73.5)	103 (68.7)	0.291
Type of surgery			
CABG	219 (59.2)	25 (16.7)	<0.001
Major procedure (other or in addition to CABG)	152 (41.1)	124 (82.7)	<0.001
Valve surgery	110 (29.7)	90 (60.0)	<0.001
Aortic surgery	22 (5.9)	28 (18.7)	<0.001
Other cardiac surgery	9 (2.4)	19 (12.7)	<0.001
Perioperative characteristics			
Perfusion time (min)	88.6 ± 55.6	126.8 ± 415.5	0.084
Aortic clamp time (min)	60.0 ± 37.9	59.2 ± 55.4	0.836
Off pump	12 (3.2)	3 (2.0)	0.443
Urgent surgery	33 (8.9)	9 (6.0)	0.268
Rethoracotomy	29 (7.8)	20 (13.3)	0.053

Dichotomous variables are shown as number (%) and continuous variables are presented as mean ± SD

Overall, 82 (15.8 %) patients suffered from postoperative infection. Of the patients with an infectious complication, 87 % had a non-surgical site infection. The occurrence of a surgical site infection was less common and was present in 13 % of patients with postoperative infection. Pneumonia was the most common infection and accounted for 59 % of all infectious complications. In 59 (72 %) patients with a postoperative infection, one or more microorganisms were detected. Characteristics of patients with and without postoperative infection are shown in Table 2.

**Table 2:** Patient characteristics according to the occurrence of postoperative infection

Variable	Infection group (*n* = 82)	Control group (*n* = 438)	*p*-value
Patient characteristics			
Sex (male)	56 (68.3)	291 (66.4)	0.744
Age (years)	70.2 ± 12.0	68.4 ± 11.0	0.189
BMI (kg/m^2^)	27.8 ± 4.9	27.3 ± 4.6	0.393
COPD	57 (69.5)	219 (50.0)	0.001
Diabetes	21 (25.6)	118 (26.9)	0.803
Congestive heart failure	41 (50.0)	174 (39.7)	0.083
Renal failure	20 (24.4)	64 (14.6)	0.027
Peripheral artery disease	18 (22.0)	80 (18.3)	0.389
Three-vessel coronary artery disease	40 (48.8)	199 (45.4)	0.494
EURO score	6.0 ± 3.5	5.2 ± 3.6	0.074
Previous myocardial infarction	27 (32.9)	177 (40.4)	0.197
Previous cardiac surgery	9 (11.0)	53 (12.1)	0.773
Preoperative medication			
Statin	45 (54.9)	325 (74.2)	0.000
Beta-blocker	58 (70.7)	326 (74.4)	0.484
ACE-inhibitor	31 (37.8)	179 (40.9)	0.604
Prednisone	15 (18.3)	44 (10.0)	0.031
Proton pump inhibitor	34 (41.5)	187 (42.7)	0.836
Aspirin	26 (31.7)	147 (33.6)	0.744
Postoperative medication			
Statin	45 (54.9)	341 (77.9)	0.000
Beta-blocker	60 (73.2)	351 (80.1)	0.143
ACE-inhibitor	36 (43.9)	200 (45.8)	0.756
Prednisone	13 (15.9)	43 (9.8)	0.109
Proton pump inhibitor	53 (64.6)	322 (73.9)	0.087
Type of surgery			
CABG	34 (41.5)	210 (47.9)	0.280
Major procedure (other or in addition to CABG)	47 (57.3)	229 (52.3)	0.402
Valve surgery	32 (39.0)	168 (38.4)	0.909
Aortic surgery	9 (11.0)	41 (9.4)	0.649
Other cardiac surgery	4 (4.9)	24 (5.5)	0.825
Perioperative characteristics			
Perfusion time (min)	106.1 ± 72.8	98.4 ± 246.9	0.781
Aortic clamp time (min)	65.6 ± 50.8	58.7 ± 42.1	0.185
Off pump	0 (0.0)	15 (3.4)	0.089
Urgent surgery	8 (9.8)	34 (7.8)	0.543
Rethoracotomy	11 (13.4)	38 (8.7)	0.180

Dichotomous variables are shown as number (%) and continuous variables are presented as mean ± SD

Patients on preoperative statin therapy suffered less frequently from postoperative infection compared with non-users (12.2 % versus 24.7 %, respectively; adjusted odds ratio (AOR): 0.329, 95 % CI: 0.19–0.57; *P* < 0.001; Table 3). Statin users had fewer non-surgical site infections compared with non-users (AOR: 0.253, 95 % CI: 0.14–0.45; *P* < 0.001). This was mainly caused by a reduction in postoperative pneumonia and urinary tract infection (Table 4). A surgical site infection occurred in 11 (3 %) patients on statin therapy, whereas non-users did not develop any. Overall, in-hospital mortality was 4.4 %; 3.2 % in patients on preoperative statin therapy and 7.3 % in non-users (*P* = 0.041).

**Table 3:** Results of multivariate analysis for the effect of statin therapy on postoperative infection rate

	OR (95 % CI)	*p*-value
Unadjusted	0.423 (0.260–0.687)	0.001
Adjusted for sex	0.412 (0.253–0.673)	<0.001
Adjusted for sex and age	0.417 (0.255–0.681)	<0.001
Adjusted for sex, age and three-vessel coronary artery disease	0.349 (0.204–0.596)	<0.001
Adjusted for sex, age, three-vessel coronary artery disease and COPD	0.318 (0.184–0.550)	<0.001
Adjusted for sex, age, three-vessel coronary artery disease, COPD and aortic clamp time more than 90 min	0.329 (0.189–0.572)	<0.001

**Table 4:** Incidence of study endpoints in statin and control group

	Statin group No. (%)	Control group No. (%)	*p*-value
All	370 (100)	150 (100)	
Postoperative infection	45 (12.2)	37 (24.7)	0.001
Surgical site infection	11 (3.0)	0 (0.0)	n.a.*
Mediastinitis	6 (1.6)	0 (0.0)	
Wound infection	5 (1.4)	0 (0.0)	
Non-surgical site infection	34 (9.2)	37 (24.7)	<0.001
Pneumonia	28 (7.6)	20 (13.3)	
Urinary tract infection	2 (0.5)	11 (7.3)	
Sepsis	3 (0.8)	5 (3.3)	
Other	1 (0.3)^§^	1 (0.3)^¶^	
In-hospital mortality	12 (3.2)	11 (7.3)	0.041

*n.a.: not available

^§^prostatitis

^¶^unknown

After surgery, statin therapy was initiated in 37 (7.1 %) patients who did not use statins prior to surgery. Postoperative initiation of statin therapy was not associated with a reduced risk of infection (AOR: 0.676, 95 % CI: 0.26–1.75; *P* = 0.422).

## Discussion

In this cohort of 520 patients undergoing cardiac surgery, preoperative statin therapy was associated with a 67 % reduced risk of a postoperative infectious complication. The reduction in postoperative infection by preoperative statin use was mainly caused by a reduction in non-surgical site infections, in particular less pneumonia and urinary tract infections.

Recently two meta-analyses studied the relationship between statins and outcome in patients undergoing high-risk cardiac procedures (e.g. CABG, valve replacement, percutaneous coronary intervention). Guay et al. included 29 randomised controlled trials in which statin therapy was compared with placebo. [10] Statin therapy reduced the risk of myocardial infarction (risk ratio (RR): 0.48; 95 % CI 0.38–0.61; *P* < 0.001) and there was a trend towards lower mortality (RR: 0.26; 95 % CI 0.06–1.02; *P* = 0.053). Another meta-analysis showed that preoperative statin therapy was associated with improved outcome after CABG, but not after aortic valve replacement. [11] However, postoperative infections were not studied in either studies. Papers investigating the effect of statins on postoperative infection in cardiac surgery patients have shown variable results. In a retrospective cohort study of 1934 patients undergoing CABG and/or valve surgery, 151 (7.8 %) patients developed an infection. [6] Preoperative statin use was associated with a risk reduction of 33 % (*P* = 0.04) for postoperative infection. The odds ratios for a non-surgical site infection such as pneumonia (OR: 0.67; 95 % CI: 0.43–1.04), urinary tract infection (OR: 0.65; 95 % CI: 0.31–1.39) or bacteraemia (OR: 0.71; 95 % CI: 0.32–1.58) tended to be less in patients on statins but did not reach statistical significance. Another retrospective cohort study from Kayani et al. in 6253 patients who underwent CABG showed statin use as being associated with a 26 % reduction in the postoperative infection rate (OR: 0.74; 95 % CI: 0.60–0.90). [12] In contrast, Mohamed et al. studied 7733 patients undergoing cardiac surgery and in that study statin users had similar infection rates to nonusers: 8.1 % versus 8.4 %, respectively (AOR: 1.08; 95 % CI: 0.89–1.31). [8] The incidence of any specific infection was similar in both groups, except for deep sternal/organ space infection (16 (0.6 %) statin users versus 54 (1.1 %) nonusers; *P* = 0.04). Importantly, postoperative pneumonia was not scored; considering the fact that pneumonia was diagnosed in 62 % of all infections in the study by Coleman et al. and 59 % in our study, excluding pneumonia could have influenced the results in the study by Mohamed et al.

We noticed a significant difference in infection type between statin users and nonusers. Patients on statin therapy had fewer non-surgical site infections, mainly caused by a reduced incidence of postoperative pneumonia and urinary tract infection. This finding is consistent with the results of previous studies. Using the United Kingdom (UK) General Practice Research Database, Schlienger et al. performed a retrospective case–control study in non-surgical patients and showed that statin therapy was associated with fewer cases of pneumonia, in particular, fatal pneumonia (OR: 0.47, 95 % CI: 0.25–0.88). [13] Van de Garde et al. showed that in patients with a history of diabetes only, statin users had a significantly reduced risk of pneumonia (OR: 0.49, 95 % CI: 0.35–0.69). [14] In contrast, in our study, surgical site infections occurred more often in patients on statin therapy than nonusers. This finding has not been described before. Known risk factors for surgical site infections, e.g. diabetes, obesity, prolonged operation time, did not appear to be more common in patients on statins in our study. Unfortunately, due to the small numbers we were not able to further investigate these effects with multivariable analysis. The same applies for potential effects of statins on mortality. Crude significant reduced in-hospital mortality could be observed in our study.

If statin therapy is associated with less postoperative non-surgical site infections, is there a reasonable explanation? It is known that statins suppress the expression of various cytokines, chemokines and adhesion molecules and modulate coagulation toward a less prothrombotic state, but is there a direct effect on bacterial growth? [5] In an experimental study, mice pretreated with low-dose simvastatin and challenged with *Staphylococcus aureus* intratracheally or intravenously had lower lung bacterial burden compared with mice that were pretreated with placebo. [15] Bacterial killing was enhanced in simvastatin-pretreated mice, while mortality was decreased. Catron et al. studied mice pretreated with lovastatin and intraperitoneally infected with *Samonella enterica*. [16] Intracellular proliferation of *Salmonella enterica* was inhibited 6- to 10-fold by lovastatin. In summary, in animals, statins seem to be bactericidal and decrease bacterial proliferation. However, well-conducted human studies remain lacking.

During hospitalisation, a patient’s drug treatment is modified frequently. For example, statin therapy can be initiated after surgery or discontinued for the remainder of the postoperative period. In a recent randomised controlled trial in patients admitted with severe sepsis, patients were randomised to atorvastatin treatment or placebo. [17] Patients in whom statin treatment was continued showed improved survival, while new-onset statin treatment did not result in improved outcome. Our study revealed that statin therapy was initiated in 37 (7 %) patients following surgery. Newly initiated statin therapy, however, was not associated with a reduced chance of developing an infection. These results may imply that the possible protective effect of statin therapy on postoperative infection is not immediately reached after the start of therapy, but that it may take weeks before a beneficial effect is apparent.

Compared with other reports, postoperative infection and mortality rates appeared to be relatively high in our study population. The most plausible explanation for this could be the fact that we applied a broad definition for postoperative infection and that we studied patients who were hospitalised more than one day before surgery. One could hypothesise that these patients represent unstable cases and that these patient have a prolonged risk of nosocomial infections. Unfortunately, we were not able to test this hypothesis in depth because of lacking information on outcomes and antibiotic exposure for the excluded patients. Nevertheless, the final population in the present study represents the population that could benefit from interventions such as initiating statins in hospital before surgery if proven beneficial.

Our study has several limitations. First, the results of our study are limited because the data were derived in a retrospective manner. Second, compared with similar studies, our study population is relatively small. And third, we did not have specific information on the length of statin therapy prior to surgery, which prevented additional analysis on timing of statin exposure.

In conclusion, this study describes a protective association between preoperative statin therapy and postoperative infectious complications in a high-risk cardiac surgical cohort. Randomised controlled trials are required to further clarify the role of statin therapy in preventing postoperative infections in cardiac surgery.
